# Supplementary material for: Phenylacetic acid metabolism in land plants: novel pathways and metabolites
Source: J Exp Bot. 2025 Mar 24;76(12):3427–43. doi: 10.1093/jxb/eraf092 (PMC12369477; doi:10.1093/jxb/eraf092)
Supplement: eraf092_suppl_Supplementary_Tables_S1-S5_Figures_S1-S3 [file eraf092_suppl_supplementary_tables_s1-s5_figures_s1-s3.pdf]

# Supplemental information

## Phenylacetic acid metabolism in land plants: novel pathways and metabolites

Pavel Hladík[1,2], Federica Brunoni[1,2], Asta Žukauskaitė[3], Marek Zatloukal[3], Jakub Bělíček[4], David Kopečný[4], Pierre Briozzo[5], Nathan Ferchaud[5], Ondřej Novák[1,2], and Aleš Pěnčík[1,2,]*

[1]*Laboratory of Growth Regulators, Faculty of Science, Palacký University, Olomouc, Czech Republic*
[2]*Laboratory of Growth Regulators, Institute of Experimental Botany, The Czech Academy of Sciences, Olomouc, Czech Republic*
[3]*Department of Chemical Biology, Faculty of Science, Palacký University, Olomouc, Czech Republic*
[4]*Department of Experimental Biology, Faculty of Science, Palacký University, Olomouc, Czech Republic*
[5]*Université Paris-Saclay, INRAE, AgroParisTech, Institute Jean-Pierre Bourgin for Plant Sciences (IJPB), 78000, Versailles, France*

** Corresponding author: ales.pencik@upol.cz*

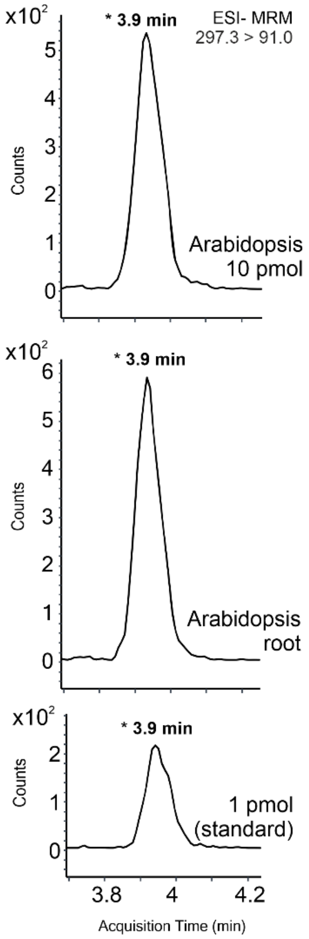


**Figure S1: Representative MRM chromatograms of PAA-glc.** Retention times of PAA-glc in 1 pmol of PAA-glc standard, 2 mg of Arabidopsis roots extract and 2 mg of Arabidopsis spiked with 10 pmol of reference standard.

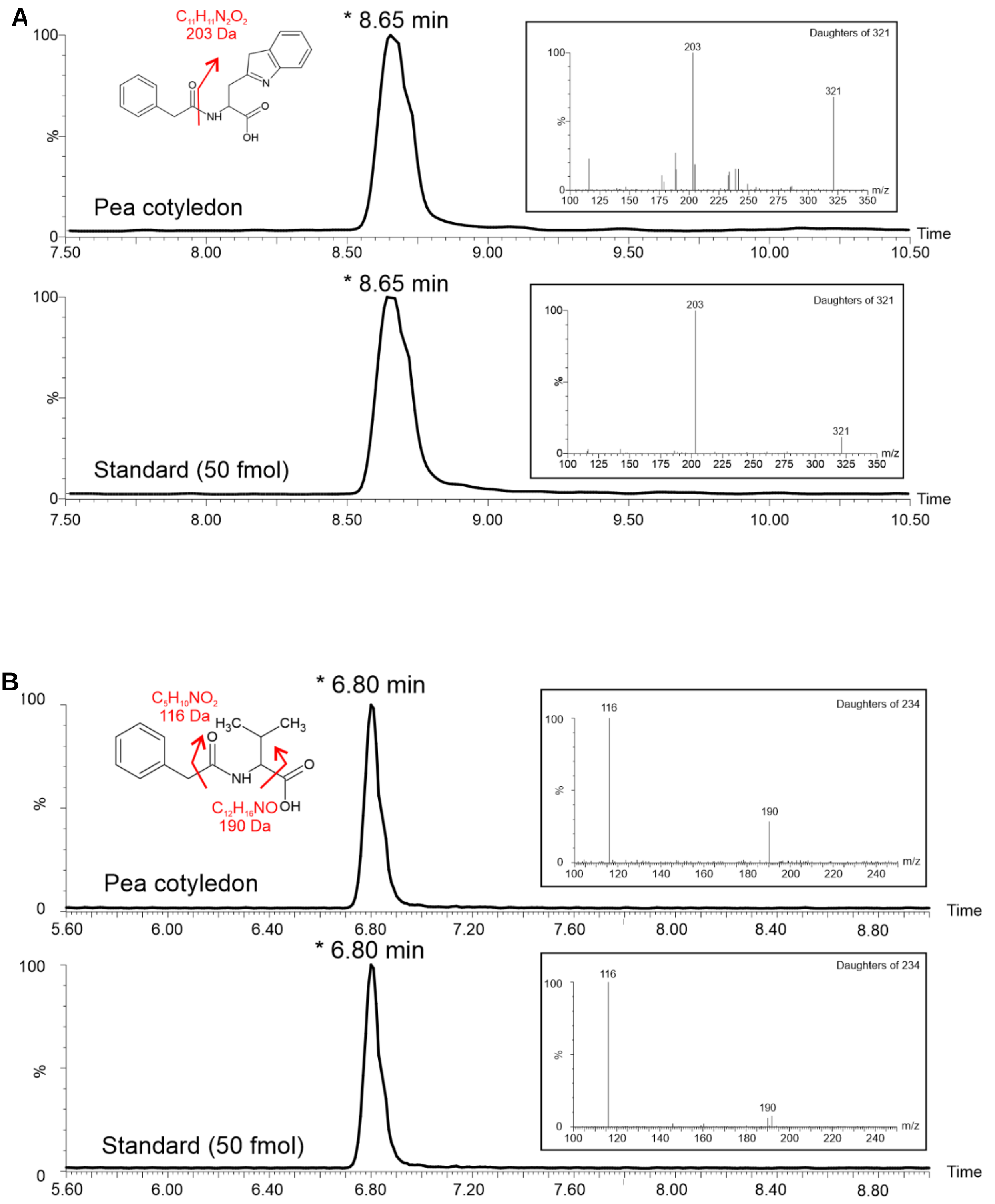


**Figure S2: PAA-Trp and PAA-Val identification.** PAA-Trp (A) and PAA-Val (B) were measured in MRM mode using 2 mg fresh weight (FW) of pea cotyledons. Retention times were compared to reference standards. For confirmation, MRM product ion confirmation (PIC) scans were performed, and fragmentation spectra are shown for both standard compounds and endogenous metabolites.

Samples were prepared as described in the Methods section. After evaporation, samples were dissolved in 30 µl of 10% methanol prior to LC-MS/MS analysis using an Acquity UPLC® System (Waters, Milford, MA, USA) coupled to a Xevo™ TQ MS triple quadrupole mass spectrometer (Waters MS Technologies, Manchester, UK). Chromatographic separation was performed on a reverse-phase Kinetex C18 100A

column (50 mm × 2.1 mm, 1.7 μm particle size; Phenomenex). The mobile phase consisted of methanol (A) and redistilled water (B), both containing 0.1% acetic acid. The gradient elution program was as follows: 0 min – 90% B, 11.5 min – 10% B, 11.75 min – 0% B, 14.75 min – 0% B, 15 min – 90% B. The total run time was 18 min with a flow rate of 0.3 ml/min. Samples were stored in an autosampler at 4°C, and the column was maintained at 30°C. An injection volume of 5 μl was used. The eluate was introduced into the electrospray ionization (ESI) source operating in negative mode (ESI−) with the following optimized conditions: source/desolvation temperature, 150/600°C; cone/desolvation gas flow, 150/1000 L/h; capillary voltage, 1 kV; cone voltage, 20 V; and collision gas flow, 0.14 ml/min. Fragmentation of PAA-Trp (collision energy [CE] 20 V) and PAA-Val (CE 15 V) was detected using MRM PIC scans. Data were processed using MassLynx v4.2 (Waters MS Technologies, Manchester, UK).

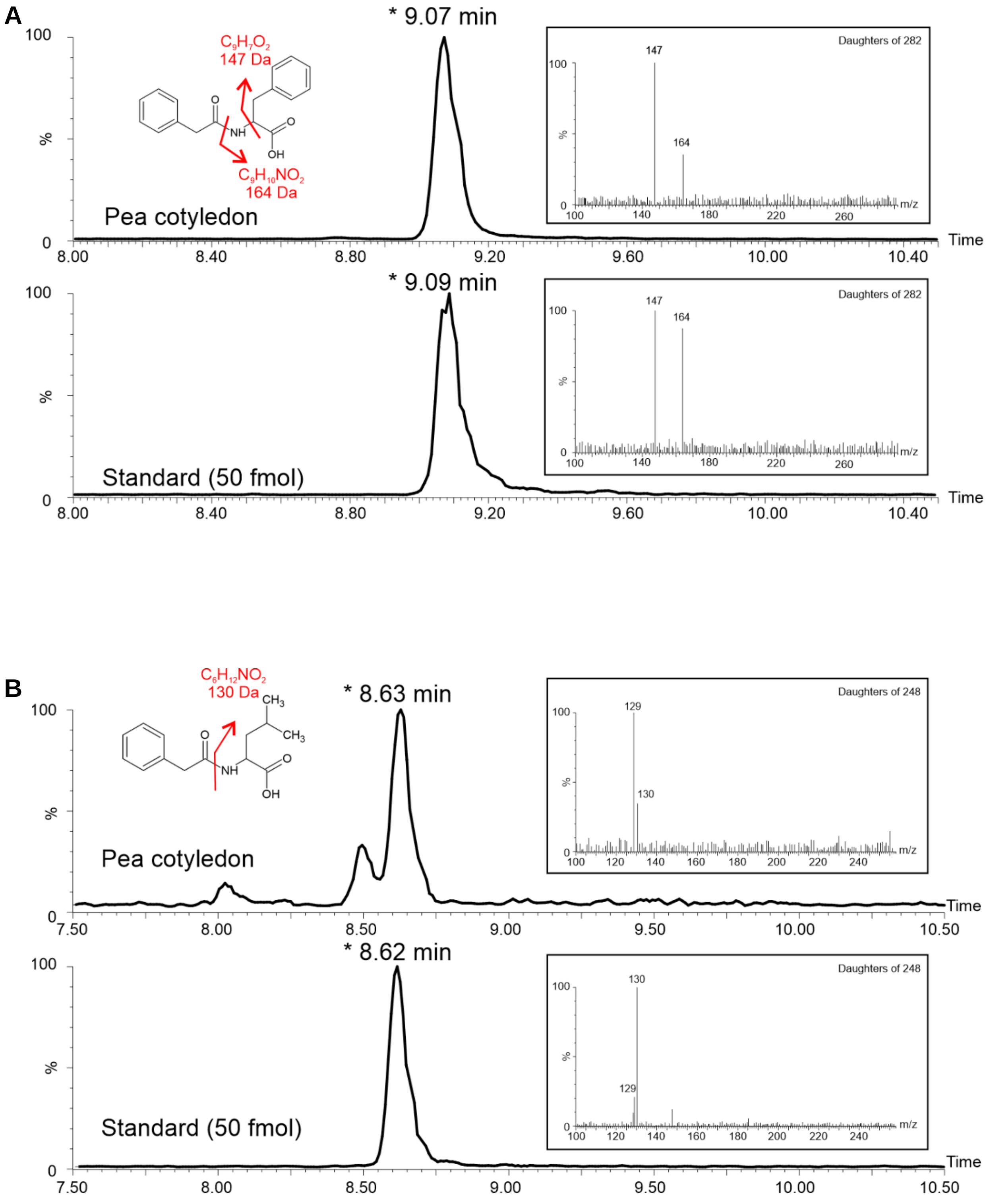


**Figure S3: PAA-Leu and PAA-Phe identification.** PAA-Phe (A) and PAA-Leu (B) were measured in MRM mode using 2 mg fresh weight (FW) of pea cotyledons. Retention times were compared to reference standards. For confirmation MRM product ion confirmation (PIC) scans were performed, and fragmentation spectra are shown for both standard compounds and endogenous metabolites. Fragmentation of PAA-Leu (CE 25 V) and PAA-Phe (CE 30 V) were analysed using MRM PIC scans due to their low concentrations in plants, which results in their levels below limit of detection during MS/MS scan. Samples were measured by LC-MS/MS conditions as described above.

**Table S1: Conditions and parameters of HPLC-MS/MS method.** For each PAA metabolite and its corresponding internal standard (IS) diagnostic MRM transition and CE were optimized. Additionally, retention time (RT), limit of detection (LOD), linear range and coefficient of determination ($R^2$) were measured and calculated. Analytes were detected by the MS instrument with optimised conditions as described: nebulizer pressure, 25 psi; drying gas flow and temperature, 14 l $min^{-1}$ and 130°C; sheath gas flow and temperature, 12 l $min^{-1}$ and 400°C; capillary voltage, 3.0 kv; nozzle voltage, 0 V.

| Compound | MRM transition | IS | MRM transition | CE (V) | retention time (min) | LOD (pmol) | linear range (pmol) | $R^2$ |
|---|---|---|---|---|---|---|---|---|
| **PAA** | 135.1 > 91.0 | [$^{13}C_6$]PAA | 141.1 > 97.0 | 2 | 5 | 4.5 $10^{-2}$ | 4.5 $10^{-2}$ - 90 | 0.9965 |
| **PAA-Asp** | 250.1 > 132.0 | [$^{13}C_6$]PAA-Asp | 256.2 > 132.0 | 10 | 2.9 | 4.5 $10^{-3}$ | 4.5 $10^{-3}$ - 90 | 0.9973 |
| **PAA-glc** | 297.3 > 91.0 | [$^{13}C_6$]PAA-Glu | 270.2 > 146.0 | 19 | 3.9 | 4.5 $10^{-2}$ | 9.0 $10^{-2}$ - 90 | 0.9904 |
| **PAA-Glu** | 264.1> 146.0 | [$^{13}C_6$]PAA-Glu | 270.2 > 146.0 | 12 | 3.7 | 9.0 $10^{-2}$ | 9.0 $10^{-2}$ - 90 | 0.996 |
| **PAA-Leu** | 248.2 > 130.1 | [$^{13}C_6$]PAA-Glu | 270.2 > 146.0 | 12 | 10.2 | 9.0 $10^{-5}$ | 9.0 $10^{-5}$ - 9 | 0.9985 |
| **PAA-Phe** | 282.2 > 164.1 | [$^{13}C_6$]PAA-Glu | 270.2 > 146.0 | 14 | 10.7 | 4.5 $10^{-4}$ | 4.5 $10^{-4}$ - 9 | 0.998 |
| **PAA-Trp** | 321.2 > 203.1 | [$^{13}C_6$]PAA-Glu | 270.2 > 146.0 | 14 | 10.2 | 4.5 $10^{-3}$ | 4.5 $10^{-3}$ - 9 | 0.9974 |
| **PAA-Val** | 234.1 > 116.1 | [$^{13}C_6$]PAA-Glu | 270.2 > 146.0 | 12 | 8.3 | 4.5 $10^{-4}$ | 4.5 $10^{-4}$ - 9 | 0.9983 |

**Table S2: Method validation in Arabidopsis extract.** Validation was conducted according to the protocol described by Hladík et al., 2023. Method accuracy (expressed as % BIAS) and precision (expressed as % RSD) were assessed through a spiking experiment. Arabidopsis seedlings (10 mg, homogenized) were extracted in 1 ml Na-phosphate buffer, and the extracts from five samples were pooled. The pooled extract was divided into 200 µl aliquots, with each aliquot spiked with 5 pmol of stable isotope-labelled standards. Unlabelled standards (1 or 10 pmol) were then added to the aliquots. The samples underwent purification using an in-tip µSPE method, and the concentrations of analytes were measured by HPLC-MS/MS with isotope dilution. Additionally, a separate set of plant extracts was processed without unlabelled standards, allowing for the subtraction of endogenous auxin metabolite levels to calculate recovery rates. Each sample was analysed in five replicates.

| **Analyte** | **1 pmol** | | | **10 pmol** | | |
|---|---|---|---|---|---|---|
| | pmol | BIAS (%) | RSD (%) | pmol | BIAS (%) | RSD (%) |
| **PAA** | 1.15 ± 0.42 | **-15** | **37** | 10.13 ± 0.68 | **-1** | **7** |
| **PAA-Asp** | 1.07 ± 0.11 | **-7** | **10** | 10.21 ± 0.30 | **-2** | **3** |
| **PAA-Glu** | 0.95 ± 0.15 | **5** | **16** | 9.60 ± 0.14 | **4** | **1** |
| **PAA-Val** | 0.92 ± 0.03 | **8** | **3** | 10.58 ± 0.34 | **-6** | **3** |
| **PAA-Leu** | 0.91 ± 0.05 | **1** | **5** | 11.25 ± 0.29 | **-13** | **3** |
| **PAA-Phe** | 0.97 ± 0.05 | **3** | **5** | 10.89 ± 0.33 | **-9** | **3** |
| **PAA-Trp** | 0.93 ± 0.03 | **7** | **3** | 11.09 ± 0.47 | **-11** | **4** |
| **PAA-glc*** | 8.46 ± 2.06 | **15** | **24** | 48.29 ± 1.54 | **3** | **5** |

* PAA-glc was spiked with 10 and 50 pmol

**Table S3: Method validation in pea extract.** Validation was conducted according to the protocol described by Hladík et al., 2023. Method accuracy (expressed as % BIAS) and precision (expressed as % RSD) were assessed through a spiking experiment. Pea seedlings (10 mg, homogenized) were extracted in 1 ml Na-phosphate buffer, and the extracts from five samples were pooled. The pooled extract was divided into 200 µl aliquots, with each aliquot spiked with 5 pmol of stable isotope-labelled standards. Unlabelled standards (1 or 10 pmol) were then added to the aliquots. The samples underwent purification using an in-tip µSPE method, and the concentrations of analytes were measured by HPLC-MS/MS with isotope dilution. Additionally, a separate set of plant extracts was processed without unlabelled standards, allowing for the subtraction of endogenous auxin metabolite levels to calculate recovery rates. Each sample was analysed in five replicates

| **Analyte** | **1 pmol** | | | **10 pmol** | | |
|---|---|---|---|---|---|---|
| | pmol | BIAS (%) | RSD (%) | pmol | BIAS (%) | RSD (%) |
| **PAA** | 1.18 ± 0.17 | **-17** | **14** | 8.33 ± 0.16 | **17** | **2** |
| **PAA-Asp** | 1.02 ± 0.12 | **-2** | **12** | 9.52 ± 0.39 | **5** | **4** |
| **PAA-Glu** | 0.98 ± 0.07 | **2** | **7** | 9.45 ± 0.29 | **6** | **3** |
| **PAA-Val** | 0.71 ± 0.03 | **29** | **4** | 7.55 ± 0.53 | **25** | **7** |
| **PAA-Leu** | 1.05 ± 0.04 | **-5** | **4** | 10.65 ± 0.62 | **-6** | **6** |
| **PAA-Phe** | 1.00 ± 0.07 | **0** | **7** | 10.58 ± 0.49 | **-6** | **5** |
| **PAA-Trp** | 0.88 ± 0.06 | **12** | **6** | 10.29 ± 0.80 | **-3** | **8** |
| **PAA-glc*** | 8.16 ± 0.40 | **18** | **5** | 36.65 ± 2.32 | **27** | **6** |

* PAA-glc was spiked with 10 and 50 pmol

**Table S4: Data collection and refinement statistics.**

| Enzyme | AtGH3.6 | |
|---|---|---|
| PDB ID | **9FXD** | **9FWD** |
| Ligand | Asp + AMP | AMP |
| Space group | $P6_4$ | $P6_4$ |
| Asymmetric unit | 2 monomers | 2 monomers |
| Unit cell (Å) | | |
| a | 197.9 | 197.0 |
| b | 197.9 | 197.0 |
| c | 65.3 | 65.2 |
| $\alpha$ (°) | 90.0 | 90.0 |
| $\beta$ (°) | 90.0 | 90.0 |
| $\gamma$ (°) | 120.0 | 120.0 |
| Diffraction limits by STARANISO (Å) | 2.13/2.13/1.74 | 2.61/ 2.61/1.93 |
| Resolution (Å)[a] | 98.9–1.74 (1.97-1.74) | 85.3–1.93 (1.97-1.74) |
| Observed reflections | 1474201 (59485) | 1224027 (50777) |
| Unique reflections | 96838 (4844) | 58596 (2932) |
| Completeness spherical (%) | 64.2 (10.3) | 53.5 (8.7) |
| Completeness ellipsoidal (%) | 96.0 (70.3) | 95.4 (76.6) |
| $I/\sigma(I)$ | 11.3 (1.9) | 14.6 (2.0) |
| $R_{sym}$ | 0.161 (1.429) | 0.174 (1.727) |
| $R_{meas}$ | 0.166 (1.490) | 0.179 (1.776) |
| $R_{pim}$ | 0.043 (0.421) | 0.039 (0.411) |
| $CC_{1/2}$[b] | 99.9 (70.5) | 99.8 (63.7) |
| Amino acid residues | 1170 | 1170 |
| Water molecules | 676 | 569 |
| $R_{cryst}$ (%) | 0.1976 | 0.2027 |
| $R_{free}$ (%)[c] | 0.2215 | 0.2402 |
| RMSD bond lengths (Å) | 0.009 | 0.008 |
| RMSD bond angles (°) | 1.06 | 1.01 |
| Mean B value (Å$^2$): | | |
| overall | 33.5 | 44.5 |
| protein chains (A/B) | 32.9/34.1 | 43.1/46.4 |
| water molecules | 34.3 | 40.2 |
| AMP (A/B) | 18.2/21.3 | 28.2/32.3 |
| Asp (A/B) | 33.9/35.0 | -/- |
| Ramachandran statistics (%)[d]: | | |
| favored | 98.9 | 98.4 |
| outliers | 0.0 | 0.0 |
| Molprobity Clashscore[d] | 1.99 | 1.45 |
| Molprobity Overall score[d] | 1.01 | 0.88 |

[a] Numbers in parentheses represent values in the highest resolution shell.

[b] $CC_{1/2}$ stands for a percentage of correlation between intensities from a random half-dataset.

[c] The 5% test set.

[d] Generated with MolProbity (Chen et al 2010).

**Table S5: Ligand Lead Finder rank (LF), docking score and ΔG (Gibbs free energy) score for selected amino acid substrates and products.** Docking was performed in FLARE (https://www.cresset-group.com).

| | AtGH 3.5 | | AtGH 3.6 | |
|---|---|---|---|---|
| **Ligand** | LF Rank score | LF ΔG kcal·mol$^{-1}$ | LF Rank score | LF ΔG kcal·mol$^{-1}$ |
| Asp | −5.315 | −7.560 | −6.615 | −7.101 |
| Glu | −5.164 | −7.285 | −6.669 | −7.611 |
| IAA-Asp | −8.795 | −7.361 | −9.786 | −8.563 |
| IAA-Glu | −7.841 | −6.992 | −9.510 | −7.842 |
| PAA-Asp | −7.107 | −6.800 | −8.560 | −7.530 |
| PAA-Glu | −7.235 | −6.392 | −8.662 | − 7.521 |

## Supplemental references

**Chen, V.B., Arendall, W.B. 3rd, Headd, J.J., Keedy, D.A., Immormino, R.M., Kapral, G.J., Murray, L.W., Richardson, J.S., Richardson, D.C.** (2010). MolProbity: all-atom structure validation for macromolecular crystallography. *Acta Crystallogr D Biol Crystallogr.* **66**: 12-21.

**Hladík, P., Petřík, I., Žukauskaitė, A., Novák, O., Pěnčík, A.** (2023). Metabolic profiles of 2-oxindole-3-acetyl-amino acid conjugates differ in various plant species. *Frontiers in Plant Science* **14**: 1217421.
